# Commentary on “Protective Effect of Regular Physical Activity Against Diabetes‐Related Lower Extremity Amputation”

**DOI:** 10.1111/1753-0407.70055

**Published:** 2025-01-22

**Authors:** Zeinab Mohseni Afshar, Mohammad Barary, Arefeh Babazadeh, Fatemeh Rasulpur, Soheil Ebrahimpour

**Affiliations:** ^1^ Infectious Diseases and Tropical Medicine Research Center, Health Research Institute Babol University of Medical Sciences Babol Iran; ^2^ Student Research Committee, School of Medical Education and Learning Technologies Shahid Beheshti University of Medical Sciences Tehran Iran; ^3^ Student Research Committee Babol University of Medical Sciences Babol Iran

Dear Editor,

We read with great interest the article titled “Protective effect of regular physical activity against diabetes‐related lower extremity amputation,” published in your esteemed journal [1]. This study's objective—to evaluate the protective effects of appropriate and regular physical activity (PA) on the risk of lower extremity amputation (LEA) in individuals with diabetes—is both timely and relevant. We commend the authors for their valuable contributions to this critical area of research. However, certain study aspects warrant further discussion to enhance their scientific rigor and applicability.

First, the study did not incorporate specific laboratory parameters that could have strengthened its conclusions. Including biomarkers such as albumin, hemoglobin, thyroid and liver function tests, Vitamin D, Vitamin B12, Vitamin B3, neutrophil‐to‐lymphocyte ratio (NLR), and platelet‐to‐lymphocyte ratio (PLR) would provide a more comprehensive understanding of the patients' risk profiles and the potential mechanisms linking PA to reduced LEA risk [2]. For instance, NLR and PLR are well‐established predictors of mortality and complications in diabetic foot ulcers and could offer additional prognostic value.

Second, the study lacked detailed information regarding medications other than antidiabetic agents. Drugs such as antihypertensives and lipid‐lowering therapies can significantly impact vascular health and diabetic outcomes. Without this data, it is challenging to isolate PA's protective effects fully.

Third, while the study considered some comorbidities, a broader exploration of underlying conditions is necessary. Comorbidities such as malignancies, psychological disorders, cerebrovascular diseases, bone deformities, diabetic retinopathy, and autoimmune diseases—in addition to diabetic neuropathy and peripheral artery disease (PAD)—may influence both PA participation and LEA risk [3]. Addressing these factors would provide a clearer understanding of the interactions between diabetes‐related complications and PA.

Moreover, the study would benefit from additional demographic information, including educational level, urban versus rural residency, and history of previous diabetic ulcers. These variables are crucial as they can significantly influence diabetes management and adherence to PA regimens. Furthermore, there was no discussion regarding insulin resistance or the results of venous examinations, both pertinent to evaluating the risk of LEA.

In conclusion, while this study highlights the protective role of regular PA in preventing LEA among individuals with diabetes, addressing the limitations mentioned above would enhance its robustness and clinical relevance. Such considerations are critical for healthcare providers as they develop tailored strategies to improve patient outcomes. We hope the esteemed authors and editorial board will consider these constructive critiques and provide justifications or clarifications for the concerns raised.

## Author Contributions

**Zeinab Mohseni Afshar:** conceptualization, methodology. **Mohammad Barary:** writing – original draft, writing – review and editing. **Arefeh Babazadeh:** investigation, writing – original draft. **Fatemeh Rasulpur:** investigation, writing – original draft. **Soheil Ebrahimpour:** investigation, supervision, writing – original draft.

## Ethics Statement

No ethical approval was required as this letter‐to‐the‐editor article has no original research data.

## Conflicts of Interest

The authors declare no conflicts of interest.

## Data Availability

Data sharing is not applicable to this article as no new data were created or analyzed in this study.
